# Medically unexplained physical symptoms and work functioning over 2 years: their association and the influence of depressive and anxiety disorders and job characteristics

**DOI:** 10.1186/s12875-016-0443-x

**Published:** 2016-04-14

**Authors:** Madelon den Boeft, Jos W. R. Twisk, Trynke Hoekstra, Berend Terluin, Brenda W. J. H. Penninx, Johannes C. van der Wouden, Mattijs E. Numans, Henriette E. van der Horst

**Affiliations:** Department of General Practice & Elderly Care Medicine, EMGO Institute for Health and Care Research, VU University Medical Center, Amsterdam, The Netherlands; Department of Epidemiology & Biostatistics, EMGO Institute for Health and Care Research, VU University Medical Center, Amsterdam, The Netherlands; Department of Psychiatry, EMGO Institute for Health and Care Research, VU University Medical Center, Amsterdam, The Netherlands; Department of Public Health and Primary Care, Leiden University Medical Center, Leiden, The Netherlands

**Keywords:** Medically unexplained physical symptoms, Work functioning, Disability, Absenteeism, Psychiatric disorders

## Abstract

**Background:**

Medically unexplained physical symptoms (MUPS) are highly prevalent and may affect work functioning. In this study we aimed to assess the longitudinal association between MUPS and work functioning over 2 years and the influence of job characteristics and depressive and anxiety disorders on this association.

**Methods:**

We assessed the longitudinal association between MUPS and work functioning, operationalized in terms of absenteeism and disability at work, in 1887 working participants from the Netherlands Study of Depression and Anxiety (NESDA). The NESDA study population included participants with a current depressive and/or anxiety disorder, participants with a lifetime risk and/or subthreshold symptoms and healthy controls. Absenteeism was assessed with the Health and Labour Questionnaire Short Form and disability with the World Health Organization Disability Assessment Schedule II. MUPS were measured with the Four Dimensional Symptom Questionnaire. Measurements were taken at baseline and at 2 years follow-up. We used mixed model analyses to correct for the dependency of observations within participants.

**Results:**

MUPS were positively associated with disability (regression coefficient 0.304; 95 % CI 0.281–0.327) and with short and long-term absenteeism over 2 years (OR 1.030, 95 % CI 1.016–1.045; OR 1.099, 95 % CI 1.085–1.114). After adjusting for depressive disorders, anxiety disorders and job characteristics, associations weakened but remained significant.

**Conclusion:**

Our results show that MUPS were positively associated with disability and absenteeism over 2 years, even after adjusting for depressive and anxiety disorders and job characteristics. This suggests that early identification of MUPS and adequate management is important.

## Background

Medically unexplained physical symptoms (MUPS) are often presented to both primary and secondary health care physicians and may have major impact on daily functioning [1, 2]. MUPS are physical symptoms not satisfactorily explained by a somatic underlying condition after an adequate medical examination and represent a broad spectrum of symptoms in varying degrees of severity [3, 4].

It is known that MUPS interfere with work functioning, both in terms of absenteeism and disability [5–7]. Not being able to work or not performing at work optimally is not only a burden for patients and their direct environment, but also for the community due to increasing costs [8, 9].

In the Netherlands, studies among workers have shown that high levels of somatic symptoms and distress are determinants for prolonged absenteeism and enduring disabilities [6, 10, 11]. It was also shown that the prevalence of severe MUPS was higher in the long-term absent employees compared to the non-sick working population [7]. These findings are supported by international studies [12, 13] and the relevance of the problem is reinforced by a 10-year follow-up study by Rask et al., who concluded that not only severe MUPS have significant impact on work functioning, but also mild and recent onset MUPS [5]. Despite the relevance of absenteeism and disability from work caused by MUPS, limited research has been performed to assess potential influencing factors on the relationship between MUPS and work functioning and their association over time [14].

It is known that unfavourable job characteristics, such as long working hours and low occupational status, can influence someone’s functioning negatively. Lower graded jobs are associated with more absenteeism and disability, compared to higher graded jobs [15]. The same applies to high demands, whether or not in combination with low support by colleagues, low task control [16–18] and long working hours [19]. The question arises to what extent this also applies to MUPS. Furthermore, as we know that MUPS are often accompanied by depressive and anxiety disorders, it is important to understand the influence of comorbidity of depressive and/or anxiety disorders on work functioning over time in patients considered having MUPS [20].

Deeper insight into the association between MUPS and work functioning and influencing factors such as job characteristics and comorbid psychiatric disorders can assist physicians in the early identification of patients at risk and it may provide opportunities to develop adequate management and prevention strategies. Therefore, in this longitudinal study we first assessed the association between MUPS and work functioning, both absenteeism and disability at work, over 2 years. Second, we investigated the influence of job characteristics and depressive and/or anxiety disorders as potential confounders in this association.

## Methods

### Design and study population

The present longitudinal analysis is part of the Netherlands Study of Depression and Anxiety (NESDA). The NESDA is a multisite naturalistic cohort study, which aims to describe the long-term course and consequences of depressive and anxiety disorders and to examine its predictors. A detailed description of the design and sampling is provided elsewhere [21]. In summary, 2981 participants between 18 and 65 years were included. The NESDA study population consisted of participants with current depressive and/or anxiety disorders, participants with a lifetime risk or subthreshold symptoms and healthy controls. Recruitment of participants took place in primary care practices (*n* = 1610), outpatient mental health care institutions (*n* = 807) and in the general population (*n* = 564). Exclusion criteria were not being fluent in the Dutch language or having a primary diagnosis of a psychotic, obsessive, bipolar or severe substance abuse disorder. Baseline data (T0) were collected between 2004 and 2007. The research protocol was approved by the ethical committees of participating universities and performed in accordance with the ethical standards of the Declaration of Helsinki. All participants provided written informed consent.

For the present study, we selected working participants (*n* = 1887), i.e. participants who had a paid job for more than 8 h a week at baseline (T0). Of them, 1665 (88.2 %) completed the questionnaires regarding work functioning and MUPS and were therefore included in this study (591 male, 1074 female). After 2 years (T1), 1455 (87.4 %) participants had a follow up assessment.

### Work functioning

We conceptualized work functioning in terms of disability at work and absenteeism from work.

Disability at work was assessed with the validated World Health Organization Disability Assessment Schedule II (WODAS-II) [22]. This is a 36-item instrument, assessing functioning and disability and focusing on six subscales regarding several domains in life. We focused on the domain of work activities which includes four items: 1) difficulty in day-to-day work, 2) difficulty in doing most important work tasks well, 3) difficulty in getting all work done and 4) difficulty in getting work done as quickly as needed. Respondents rated difficulty on a 5-point scale, ranging from 1 (none) to 5 (extreme, cannot do). Therefore the summated work domain scale has a range from 4 to 20 with higher scores representing a higher level of disability.

Absenteeism from work was assessed with the validated Health and Labour Questionnaire Short Form [23] and was computed by dividing the number of absent days from work during the past 6 months because of health problems by the number of actual work days per week. As this variable does not meet normality assumptions, we categorized it into three categories: no absenteeism, short-term absenteeism (<2 weeks) and long-term absenteeism (2 weeks or longer), cf. Plaisier et al. [24]. The latter two categories made a distinction possible between short-term absenteeism, probably due to self-limiting conditions such as common colds, and long-term absenteeism, which could indicate more chronic conditions.

### Medically unexplained physical symptoms

MUPS were measured with the somatization scale of the validated Four Dimensional Symptoms Questionnaire (4DSQ), a self-report questionnaire developed to measure distress, depression, anxiety and somatization as separate dimensions in primary care (25). The somatization scale comprises 16 items, all physical symptoms. The response categories on a 5-point Likert scale are worded as follows: “no”, “sometimes”, “regularly”, “often” and “very often or constantly”. In order to arrive at scale scores, the responses are scored as 0 for “no”, 1 for “sometimes” and 2 for “regularly”, “often” and “very often or constantly” and the item scores were summated into a scale score, as done by Terluin et al. with a range of 0–32 [25], indicating that MUPS is considered to be a summation of physical symptoms. Additionally, in order to facilitate clinical use and to overcome the fact that there is no linear relationship between MUPS and work functioning, we repeated the analyses with a dichotomized scale using 11 points as a clinical cut-off score, since a score of 11 or higher is considered to indicate MUPS [25].

### Job characteristics

Job characteristics were conceptualized as working hours, occupational status and psychosocial working conditions. The occupational status variable was created using the occupational categories provided by Statistics Netherlands and additional self-reported information on employment status. The original eleven categories of Statistics Netherlands were recoded into five categories: 1) high graded non-manual workers, 2) medium or low skilled non-manual workers, 3) self-employed, 4) high skilled manual workers and 5) medium or low skilled manual workers, as was done by Plaisier et al. [26]. Psychosocial working conditions consist of job demands, job control and job support and were measured with a questionnaire consisting of dichotomous items, based on the demands/control model [27]. Data on job characteristics were only gathered at baseline (T0). Unfortunately for 30 % of the participants no information regarding job characteristics could be obtained.

### Depressive and/or anxiety disorders

The presence of depressive disorders, including major depressive disorder and dysthymia as well as anxiety disorders, including generalized anxiety disorder, panic disorder with or without agoraphobia, social phobia and/or agoraphobia without panic disorder, were diagnosed with the validated Composite Interview Diagnostic Instrument (CIDI version 2.1). We only took into account diagnoses established during the past 6 months. Trained research staff interviewed all participants.

### Socio-demographic covariates and chronic diseases

Based on previous research on the association between MUPS and work functioning, the following covariates were considered as possible confounders: gender, age, level of education, marital status and the number of chronic diseases [5, 28]. We divided the level of education into three groups (basic, intermediate, high), derived from the standard classification of education from Statistics Netherlands [29]. Marital status was divided into two categories: participants married or living with a partner and participants not married or living without a partner. Participants were asked if they suffered from chronic diseases from each of the following categories: respiratory, cardiometabolic, musculoskeletal, digestive, neurological, endocrine and cancer. Diseases were only included if participants were currently treated with medicines or under specialist care. The number of diseases were summated into a scale score. Data on the covariates were only gathered at baseline (T0).

### Analysis

Descriptive statistics are presented as mean with standard deviation for normally distributed continuous data, median and inter-quartile range for skewed continuous variables and as numbers and percentages for dichotomous and categorical variables.

To assess the association between MUPS and work functioning over 2 years (T0, T1), we used linear mixed model analyses for disability and multinomial logistic mixed model analyses for absenteeism. After crude analyses, we performed adjusted analyses where covariates (sociodemographic variables and the number of chronic diseases) were included in the model. Next, we examined the confounding role of depressive and/or anxiety disorders and job characteristics on the adjusted association between MUPS and work functioning, first separately and then combined.

Effects were expressed as regression coefficients (for disability) and odds ratios (ORs) (for absenteeism) with 95 % confidence intervals (CI), representing the longitudinal association between MUPS and work functioning on average over time, reflecting both the within and between subject relationship [30]. For absenteeism, ‘no absenteeism’ was used as the reference category.

As depressive and anxiety disorders were overrepresented in the NESDA cohort, we added interaction terms to the analysis to assess if depressive and/or anxiety disorders modify the association between MUPS and work functioning. We used all observations in our analyses. As we used a longitudinal design, missing data did not have to be imputed [30].

The statistical analyses were performed in MLwiN (version 2.31) and Stata (version 13).

## Results

Table 1 shows the demographic and clinical characteristics of the study sample. At baseline, the mean age was 40 years and 65 % were women. The median score for MUPS was 8 at baseline and 6 at 2 years follow up (*p* < 0.001). When using the clinical cut-off point of 37 % had MUPS at T0 and 22 % had MUPS at T1.

**Table 1:** Demographic and clinical characteristics of research population

	Baseline (T0)	Two year follow up (T1)
	(*n* = 1665)	(*n* = 1455)
Socio-demographics		
Females, number (%)	1074 (64.5)	941 (64.7)
Age in years, mean (SD)	40.8 (11.7)	40.1 (11.4)
Level of education, number (%)		
Basic	68 (4.1)	56 (3.8)
Intermediate	886 (53.2)	749 (51.5)
High	711 (42.7)	650 (44.7)
Number of chronic diseases, median (IQR)	0.00 (0.00–1.00)	0.00 (0.00–1.00)
Married or with partner, number (%)	1223 (73.5)	1047 (72.0)
Medically unexplained physical symptoms		
Total score (0–32), median (IQR)	8.00 (4.00–13.00)	6.00 (3.00–10.00)
Somatisation (cut-off 11 points) (%)	621 (37.3)	322 (22.1)
Work functioning		
Absenteeism, number (%)		
No absenteeism	786 (47.2)	804 (55.3)
Short term absenteeism (<2 weeks)	508 (30.5)	467 (32.1)
Long term absenteeism (≥2 weeks)	371 (22.3)	184 (12.6)
Disability (0–20), mean (SD)	8.5 (4.5)	7.1 (3.7)
Job characteristics, median (IQR)		
Working hours	32.00 (24.00–38.00)	-
Job demands	0.40 (0.20–0.80)	-
Job control	0.79 (0.61–0.93)	-
Job support	0.75 (0.50–1.00)	-
Psychiatric comorbidity (number, %)		
Depressive disorder	572 (34.4)	285 (19.6)
Anxiety disorder	653 (39.2)	333 (22.9)

Means with standard deviations are given for normally distributed continuous variables, medians and inter-quartile ranges for skewed continuous variables and frequencies with percentages for dichotomous and categorical variables. *SD* standard deviation. *IQR* interquartile range

### The longitudinal association between MUPS and disability at work

We found a strong positive association between MUPS and disability at work on average over time (Table 2). The estimated crude regression coefficient of 0.295 for MUPS, measured with the continuous 4DSQ somatization scale, in relation to disability at work can be interpreted as follows: for every unit increase/difference in the 4DSQ, there is a 0.295 increase/difference in the severity of disability. Depressive disorders had the greatest influence on the association between MUPS and disability as the regression coefficient became smaller, followed by anxiety disorders. However, the association between MUPS and disability remained statistically significant. Job characteristics did not affect the association. For the dichotomous 4DSQ comparable results were found.

**Table 2:** Analyses results on the association between MUPS and disability at work over 2 years

	Regression coefficient (95 % CI) (with continuous 4DSQ)	Regression coefficient (95 % CI) (with dichotomous 4DSQ)
Crude	0.30 (0.27–0.32)	3.23 (2.93–3.53)
Adjusted^a^	0.30 (0.28–0.33)	3.21 (2.91–3.52)
Univariable^b^		
Anxiety disorder	0.26 (0.24–0.29)	2.68 (2.37–3.00)
Depressive disorder	0.23 (0.21–0.26)	2.39 (2.10–2.70)
Job characteristics		
Working hours	0.30 (0.28–0.33)	3.21 (2.90–3.52)
Occupational status	0.32 (0.29–0.34)	3.20 (2.83–3.57)
Job demands	0.30 (0.28–0.33)	3.05 (2.71–3.38)
Job control	0.30 (0.28–0.33)	3.00 (2.67–3.34)
Job support	0.30 (0.27–0.32)	2.91 (2.58–3.25)
Multivariable^b^	0.21 (0.18–0.24)	1.95 (1.59–2.31)

*4DSQ* four-dimensional symptom questionnaire

^a^Adjusted for age, gender, level of education, marital status, number of chronic diseases

^b^Univariable: influence of covariates separately; Multivariable: influence of covariates combined

When we examined whether the association between MUPS and disability was modified by depressive and/or anxiety disorders, we found a strong significant negative interaction for depressive disorders (*p* < 0.001) and a borderline significant negative interaction for anxiety disorders (*p* = 0.054), indicating that the association between MUPS and disability was weaker for participants with a depressive and/or anxiety disorder. For the dichotomous 4DSQ comparable results were found.

### The longitudinal association between MUPS and absenteeism from work

We found a strong positive association between MUPS and short-term absenteeism and an even stronger effect for long-term absenteeism, on average over time, both compared to the reference category ‘no absenteeism’ (Table 3). Depressive disorders had the strongest influence on the association between MUPS (with use of the continuous 4DSQ) and short-term absenteeism (37 % decrease in regression coefficient), followed by job characteristics (mainly caused by job support and job control; 33 and 37 % decrease in regression coefficient) and anxiety disorders (20 % decrease in regression coefficient). Depressive disorders also had the strongest influence on the association between MUPS and long-term absenteeism (40 % decrease in regression coefficient), followed by anxiety disorders (20 % decrease in regression coefficient). Job characteristics did not affect the association. Despite adjusting for these confounders, the association between MUPS and absenteeism remained significant. For the dichotomous 4DSQ comparable results were found.

**Table 3:** Analyses results on the association between MUPS and absenteeism from work over 2 years

	Odds ratio (95 % CI) (with continuous 4DSQ)		Odds ratio (95 % CI) (with dichotomous 4DSQ)	
	Short-term absenteeism	Long-term absenteeism	Short-term absenteeism	Long-term absenteeism
Crude	1.03 (1.02–1.05)	1.11 (1.09–1.13)	1.41 (1.19–1.67)	3.64 (3.06–4.34)
Adjusteda^a^	1.03 (1.02–1.05)	1.10 (1.09–1.11)	1.37 (1.15–1.62)	3.15 (2.62–3.78)
Univariable^b^				
Anxiety disorder	1.02 (1.01–1.04)	1.08 (1.06–1.09)	1.26 (1.05–1.50)	2.47 (2.04–2.99)
Depressive disorder	1.02 (1.01–1.03)	1.06 (1.04–1.07)	1.21 (1.02–1.45)	2.08 (1.72–2.52)
Job characteristics				
Working hours	1.03 (1.02–1.05)	1.10 (1.08–1.11)	1.37 (1.15–1.63)	3.15 (2.62–3.78)
Occupational status	1.03 (1.01–1.04)	1.10 (1.08–1.12)	1.27 (1.03–1.57)	2.81 (2.26–3.50)
Job demands	1.02 (1.01–1.04)	1.09 (1.08–1.11)	1.29 (1.07–1.56)	2.79 (2.28–3.41)
Job control	1.02 (1.01–1.03)	1.09 (1.07–1.11)	1.22 (1.01–1.48)	2.66 (2.17–3.25)
Job support	1.02 (1.01–1.04)	1.09 (1.07–1.11)	1.22 (1.01–1.48)	2.69 (2.20–3.30)
Multivariable^c^	1.01 (1.00–1.03)	1.04 (1.02–1.06)	1.03 (0.82–1.30)	1.61 (1.26–2.05)

Reference category: ‘no absenteeism’. *4DSQ* four-dimensional symptom questionnaire

^a^Adjusted for age, gender, level of education, marital status, number of chronic diseases

^b^Univariable: influence of covariates separately

^c^Multivariable: influence of covariates combined

When we examined whether the association between MUPS and absenteeism was modified by depressive and/or anxiety disorders, we only found a negative interaction for anxiety disorders and long-term absenteeism (*p* = 0.022), indicating that the association between MUPS and long-term absenteeism was weaker for participants with an anxiety disorder. We found no significant interaction for depressive disorders. For the dichotomous 4DSQ comparable results were found.

## Discussion

### Summary of results

With this study we aimed to assess the association between MUPS and work functioning over 2 years and the influence of job characteristics and depressive and anxiety disorders on this association. We showed that MUPS was positively associated with disability and absenteeism from work over 2 years, with a stronger effect for long-term absenteeism than for short-term absenteeism. Depressive and anxiety disorders weakened the association between MUPS and work functioning, but the association remained significant. Job characteristics only weakened the association between MUPS and short-term absenteeism, but again the association remained significant.

### Comparison with existing literature

Little research has been performed on MUPS and work functioning. Even though our study is not directly comparable with previously conducted research because of use of different designs and populations among other things, our study results are in line with other studies.

In a prospective study, Roelen et al. showed that various MUPS were associated with absenteeism and concluded that the more symptoms at baseline, the higher the risk of absenteeism a year later [10]. In two cross-sectional studies, Hoedeman et al. concluded that 15 % of employees on sickness leave suffered from severe MUPS and that severe MUPS was associated with four to six times more comorbid depressive and anxiety disorders. The authors used a cut-off score of 15 on the PHQ-15 for the categorisation of severe MUPS.

Furthermore, they concluded that employees with severe MUPS had a longer duration of absenteeism [6, 7]. In a 5-year follow-up study, Loengaard et al. found that MUPS patients had an increased risk of long-term absenteeism compared to healthy participants [31]. Rask et al. performed a 10-year follow-up study and found that recent onset MUPS and somatoform disorders have significant negative long-term impact on patient work functioning [5]. They also concluded that although depressive and anxiety disorders influence the association between MUPS and work functioning, both psychiatric disorders did not fully explain the effect, which corresponds with our findings.

We are not aware of earlier studies that examined the influence of specific job characteristics in patients with MUPS. However, there are some studies that describe the association of job characteristics with work functioning and mental health problems in the general working population. We found that job support and job control weakened the association between MUPS and short-term absenteeism, which is in line with North et al. and Melchior et al. [16, 18]. Christensen et al. found that absenteeism rates were higher for lower graded jobs compared to higher graded jobs, while Sparks et al. found that the number of working hours might be important for work functioning [19]. We did not find the same results for MUPS.

### Strengths and limitations

We believe our study has several methodological strengths, in particular the large sample of participants, recruited from primary care and secondary mental health care, and the longitudinal design. Also, all analyses were adjusted for the number of chronic diseases. Finally, for the assessment of depressive and anxiety disorders, structured interviews were used.

Our findings should be interpreted in the light of several limitations. First, as for all questionnaires assessing MUPS, the 4DSQ lacks clinical judgement. However, the 4DSQ highly correlates with the Patient Health Questionnaire-15 and the Symptom Checklist-90, other questionnaires widely used to measure MUPS. In our study we defined MUPS based on the score of the 4DSQ and MUPS is defined as a summation of physical symptoms which often remain unexplained after appropriate examination. It should be kept in mind that the definition of MUPS is controversial, i.e. whether it represents a specific disorder or whether it is a way of presenting different types of emotional distress. Second, time windows differed between the concepts: disability was measured over the past month and absenteeism over the past 6 months, while MUPS was measured over the past week. However, we do not believe that the results were much affected by this incongruence. In an additional analysis we found a correlation coefficient of 0.72 between MUPS at T0 and T1, indicating a quite stable pattern of MUPS over 2 years. Also the additional time lag analysis, relating MUPS at T0 to disability and absenteeism at T1, revealed more or less the same results (data not shown). Third, because job characteristics had 30 % missing values, we repeated the crude analyses in a reduced dataset with only complete cases. The results showed that job characteristics still had a weakening influence on the association MUPS and short-term absenteeism, but that this influence was weaker. Fourth, we have no information on the actual reasons for disability and absenteeism. Fifth, participants with depressive and/or anxiety disorders were oversampled in our study population. It is often assumed that the observed strong association between MUPS and work functioning is because of this oversampling. With the interaction analyses however, we showed that it is actually the other way around: that within participants without these disorders the association between MUPS and work functioning was stronger than in participants with these disorders. Therefore we wanted to emphasize that the observed associations may also hold for the general population. It should be noted that the negative interaction between MUPS and depressive/anxiety disorders is probably due to the strong association between depressive/anxiety disorders and disability.

Finally, the population at follow-up is slightly healthier than the original population. Although it is possible that those who did not complete follow-up measurements were more severely disturbed, we do not think that this highly influences the results of our study. However, if there is an influence, the magnitude of the observed association between MUPS and work functioning might be an underestimation of the real association due to this phenomena.

### Implication for clinical practice and future research

Disability and absenteeism may lead to a decreased quality of life for patients and high direct and indirect costs [8, 9, 32]. As we found that MUPS have an independent negative association with work functioning, attention should be paid to preventive measures in the work place, early identification of MUPS and adequate treatment. However, as depressive and anxiety disorders weakened the association between MUPS and work functioning, physicians should be aware of signs and symptoms of depressive or anxiety disorders.

As said, little is known about work environment and only a small number of studies assessed phenomena associated with disability and absenteeism among workers with MUPS [14]. With our analyses, we have shown that job support and job control influence the association between MUPS and mainly short-term absenteeism. More insight is needed about favourable job characteristics to develop interventions for prevention and treatment and return to work programs.

## Conclusions

Our results show that MUPS were positively associated with disability and absenteeism over 2 years, even after adjusting for depressive and anxiety disorders and job characteristics. This suggests that early identification of MUPS and adequate management is important.

### Ethics approval and consent to participate

The research protocol was approved by the ethical committees of participating universities (Amsterdam, Leiden and Groningen) and performed in accordance with the ethical standards of the Declaration of Helsinki. All participants provided written informed consent.

### Consent for publication

Not applicable.

### Availability of data

Data can be made available on request.

## Abbreviations

4DSQ: four dimensional symptoms questionnaire
CI: confidence interval
CIDI: composite interview diagnostic instrument
MUPS: medically unexplained physical symptoms
NESDA: Netherlands study of depression and anxiety
OR: odds ratio
WHO-DAS: world health organization disability assessment schedule
